# Evidence for large-scale gene-by-smoking interaction effects on pulmonary function

**DOI:** 10.1093/ije/dyw318

**Published:** 2017-01-12

**Authors:** Hugues Aschard, Martin D Tobin, Dana B Hancock, David Skurnik, Akshay Sood, Alan James, Albert Vernon Smith, Ani W Manichaikul, Archie Campbell, Bram P Prins, Caroline Hayward, Daan W Loth, David J Porteous, David P Strachan, Eleftheria Zeggini, George T O’Connor, Guy G Brusselle, H Marike Boezen, Holger Schulz, Ian J Deary, Ian P Hall, Igor Rudan, Jaakko Kaprio, James F Wilson, Jemma B Wilk, Jennifer E Huffman, Jing Hua Zhao, Kim de Jong, Leo-Pekka Lyytikäinen, Louise V Wain, Marjo-Riitta Jarvelin, Mika Kähönen, Myriam Fornage, Ozren Polasek, Patricia A Cassano, R Graham Barr, Rajesh Rawal, Sarah E Harris, Sina A Gharib, Stefan Enroth, Susan R Heckbert, Terho Lehtimäki, Ulf Gyllensten, Understanding Society Scientific Group, Victoria E Jackson, Vilmundur Gudnason, Wenbo Tang, Josée Dupuis, María Soler Artigas, Amit D Joshi, Stephanie J London, Peter Kraft

**Affiliations:** 1 Department of Epidemiology, Harvard TH Chan School of Public Health, Boston, MA, USA; 2 Program in Genetic Epidemiology and Statistical Genetics, Harvard TH Chan School of Public Health, Boston, MA, USA; 3 Genetic Epidemiology Group, Department of Health Sciences, University of Leicester, Leicester, UK; 4 National Institute for Health Research, Leicester Respiratory Biomedical Research Unit, Glenfield Hospital, Leicester, UK; 5 Behavioral and Urban Health Program, Behavioral Health and Criminal Justice Research Division, Research Triangle Institute (RTI) International, Research Triangle Park, NC, USA; 6 Division of Infectious Diseases, Brigham and Women Hospital, Harvard Medical School, Boston, MA, USA; 7 Division of Pulmonary, Critical Care and Sleep Medicine, Department of Internal Medicine, University of New Mexico School of Medicine, Albuquerque, NM, USA; 8 Department of Pulmonary Physiology and Sleep Medicine, Sir Charles Gairdner Hospital, Nedlands, Australia; 9 School of Medicine and Pharmacology, University of Western Australia, Crawley, Australia; 10 Icelandic Heart Association, Kopavogur, Iceland; 11 Faculty of Medicine, University of Iceland, Reykjavik, Iceland; 12 Center for Public Health Genomics, University of Virginia, Charlottesville, VA, USA; 13 Department of Public Health Sciences, Division of Biostatistics and Epidemiology, University of Virginia, Charlottesville, VA, USA; 14 Centre for Genomic & Experimental Medicine, Institute of Genetics & Molecular Medicine, University of Edinburgh, Edinburgh, UK; 15 Generation Scotland, Centre for Genomic and Experimental Medicine, University of Edinburgh, Edinburgh, UK; 16 Department of Human Genetics, Wellcome Trust Sanger Institute, Hinxton, UK; 17 MRC Human Genetics Unit, Institute of Genetics and Molecular Medicine, University of Edinburgh, Edinburgh, UK; 18 Department of Epidemiology, Erasmus Medical Center, Rotterdam, The Netherlands; 19 Population Health Research Institute, St George’s University of London, London, UK; 20 The National Heart, Lung, and Blood Institute’s Framingham Heart Study, Framingham, MA, USA; 21 The Pulmonary Center, Department of Medicine, Boston University School of Medicine, Boston, MA, USA; 22 Department of Respiratory Medicine, Ghent University Hospital, Ghent, Belgium; 23 Department of Respiratory Medicine, Erasmus Medical Center, Rotterdam, The Netherlands; 24 University of Groningen, University Medical Center Groningen, Department of Epidemiology, Groningen, The Netherlands; 25 University of Groningen, University Medical Center Groningen, Groningen Research Institute for Asthma and COPD, Groningen, The Netherlands; 26 Institute of Epidemiology I, Helmholtz Zentrum München, German Research Center for Environmental Health, Neuherberg, Germany; 27 Comprehensive Pneumology Center Munich (CPC-M), Member of the German Center for Lung Research, Munich, Germany; 28 Centre for Cognitive Ageing and Cognitive Epidemiology, University of Edinburgh, Edinburgh, UK; 29 Department of Psychology, University of Edinburgh, Edinburgh, UK; 30 Division of Respiratory Medicine, University of Nottingham, Queen’s Medical Centre, Nottingham, UK; 31 Centre for Global Health Research, Usher Institute of Population Health Sciences and Informatics, University of Edinburgh, Edinburgh, UK; 32 Department of Public Health, University of Helsinki, Helsinki, Finland; 33 Institute for Molecular Medicine, University of Helsinki, Helsinki, Finland; 34 National Institute for Health and Welfare, Department of Health, Helsinki, Finland; 35 MRC Epidemiology Unit, University of Cambridge School of Clinical Medicine, Cambridge, UK; 36 Institute of Metabolic Science, Biomedical Campus, Cambridge, UK; 37 Department of Clinical Chemistry, Fimlab Laboratories, Tampere, Finland; 38 Department of Clinical Chemistry, University of Tampere School of Medicine, Tampere, Finland; 39 Department of Epidemiology and Biostatistics, MRC–PHE Centre for Environment & Health, School of Public Health, Imperial College London, UK; 40 Center for Life Course Epidemiology, Faculty of Medicine, University of Oulu, Oulu, Finland; 41 Biocenter Oulu, University of Oulu, Oulu, Finland; 42 Unit of Primary Care, Oulu University Hospital, Oulu, Finland; 43 Department of Clinical Physiology, University of Tampere and Tampere University Hospital, Tampere, Finland; 44 Brown Foundation Institute of Molecular Medicine, University of Texas Health Science Center at Houston, Houston, TX, USA; 45 Faculty of Medicine, University of Split, Split, Croatia; 46 Division of Nutritional Sciences, Cornell University, Ithaca, NY, USA; 47 Department of Healthcare Policy and Research, Weill Cornell Medical College, NY, NY, USA; 48 Departments of Medicine and Epidemiology, Columbia University Medical Center; 49 Institute of Genetic Epidemiology, Helmholtz Zentrum München, German Research Center for Environmental Health, Neuherberg, Germany; 50 Research Unit of Molecular Epidemiology, Helmholtz Zentrum München, German Research Center for Environmental Health, Neuherberg, Germany; 51 Institute of Epidemiology II, Helmholtz Zentrum München, German Research Center for Environmental Health, Neuherberg, Germany; 52 Computational Medicine Core at Center for Lung Biology, Division of Pulmonary & Critical Care Medicine, University of Washington, Seattle, WA; 53 Department of Immunology, Genetics and Pathology, Uppsala Universitet, Science for Life Laboratory, Uppsala, Sweden; 54 Cardiovascular Health Research Unit and Department of Epidemiology, University of Washington, Seattle, WA, USA; 55 Boehringer Ingelheim Pharmaceuticals, Inc., Ridgefield, CT, USA; 56 Department of Biostatistics, Boston University School of Public Health, Boston, MA, USA; 57 Division of Gastroenterology, Massachusetts General Hospital, Boston, MA, USA. Human Services, Research Triangle Park, NC, USA; 58 Epidemiology Branch, National Institute of Environmental Health Sciences, National Institutes of Health, US Department of Health and Human Services, Research Triangle Park, NC, USA

**Keywords:** FEV_1_/FVC, smoking, gene–environment interaction, genetic risk score

## Abstract

**Background:** Smoking is the strongest environmental risk factor for reduced pulmonary function. The genetic component of various pulmonary traits has also been demonstrated, and at least 26 loci have been reproducibly associated with either FEV_1_ (forced expiratory volume in 1 second) or FEV_1_/FVC (FEV_1_/forced vital capacity). Although the main effects of smoking and genetic loci are well established, the question of potential gene-by-smoking interaction effect remains unanswered. The aim of the present study was to assess, using a genetic risk score approach, whether the effect of these 26 loci on pulmonary function is influenced by smoking.

**Methods:** We evaluated the interaction between smoking exposure, considered as either ever vs never or pack-years, and a 26-single nucleotide polymorphisms (SNPs) genetic risk score in relation to FEV_1_ or FEV_1_/FVC in 50 047 participants of European ancestry from the Cohorts for Heart and Aging Research in Genomic Epidemiology (CHARGE) and SpiroMeta consortia.

**Results:** We identified an interaction (*β_int_* = –0.036, 95% confidence interval, –0.040 to –0.032, *P* = 0.00057) between an unweighted 26 SNP genetic risk score and smoking status (ever/never) on the FEV_1_/FVC ratio. In interpreting this interaction, we showed that the genetic risk of falling below the FEV**_1_**/FVC threshold used to diagnose chronic obstructive pulmonary disease is higher among ever smokers than among never smokers. A replication analysis in two independent datasets, although not statistically significant, showed a similar trend in the interaction effect.

**Conclusions:** This study highlights the benefit of using genetic risk scores for identifying interactions missed when studying individual SNPs and shows, for the first time, that persons with the highest genetic risk for low FEV_1_/FVC may be more susceptible to the deleterious effects of smoking.

**Key Messages**

- Spirometric measures of pulmonary function are influenced by both smoking and genetics. This paper reports a genetic risk score-by-ever smoking interaction on FEV_1_/FVC (forced expiratory volume in 1 second/forced vital capacity).
- In individuals of European ancestry, the reduction in FEV_1_/FVC as a result of smoking was greater among individuals who are genetically predisposed to lower FEV_1_/FVC ratio.
- Genetic risk score-by-ever smoking interaction can allow the identification of subgroups in the population whose genetic background makes them more susceptible to the deleterious effects of smoking.

## Introduction

Spirometric measures of pulmonary function, such as the forced expiratory volume in 1 second (FEV_1_) or its ratio with the forced vital capacity (FEV_1_/FVC), form the basis of the diagnosis of chronic obstructive pulmonary disease (COPD).^1–3^ Pulmonary function measures are also used clinically to monitor severity and control of asthma and other respiratory diseases and are independent risk factors for mortality.^1–3^ Pulmonary function is strongly influenced by cigarette smoking and by multiple low-penetrance genetic variants. Indeed, genome-wide association studies (GWAS) of marginal genetic effects (i.e. not including interaction effects between genetic variants and smoking) have identified at least 26 loci associated with FEV_1_ or FEV_1_/FVC in the general population.^4^ However, the interplay between genetic factors and environmental exposures has not been well established for pulmonary function or its associated traits. More broadly, although considerable efforts have been made to identify interaction effects between genetic variants and environmental exposures across the wide range of human traits and diseases,^5^^,^^6^ such investigations have been mostly unsuccessful in detecting robust gene–environment interactions.^5^^,^^7^ The well-established effect of cigarette smoking on numerous human health outcomes^8^ makes it a serious candidate for identification of novel gene–environment interactions, especially for pulmonary traits.

Hypothesizing the presence of single nucleotide polymorphism (SNP)-by-smoking interaction, Hancock *et al.*^9^ performed a genome-wide interaction study of pulmonary function, modelling single SNP main effects and their interactions with smoking in 50 047 participants of European ancestry across 19 studies within the Cohorts for Heart and Aging Research in Genomic Epidemiology (CHARGE)^10^ and SpiroMeta consortia^11^—the largest genome-wide interaction study of pulmonary function as modified by smoking to date. However, rather than focusing on the interaction effects per se, they performed a meta-analysis of the joint test of SNP main effects and SNP-by-smoking interaction effects to improve power for identifying genetic variants associated with pulmonary function.^12^^,^^13^ Although they reported new candidate variants based on this joint test, the study did not identify any SNPs with genome-wide significant interaction with smoking.

Here, we explored gene-by-smoking interaction effects limited to genetic variants previously found to be associated with pulmonary function in standard marginal effects GWAS,^4^ therefore not including the new variants reported by Hancock *et al.*^9^ based on the joint test of main effects plus interaction. Specifically, we aimed to determine whether smoking modifies the effect of established genetic variants when considered singly or in combination using a genetic risk score summarizing the genetic predisposition to abnormal pulmonary function. The primary motivation for using genetic risk score is statistical power.^14^^,^^15^ Indeed, several genetic risk score-by-exposure interactions have already been identified in cases where single SNPs did not show evidence for statistically significant interactions.^16–21^ Genetic risk score-by-exposure interaction testing expands on the principle of omnibus test while leveraging the assumption that, for a given choice of coded alleles, most interaction effects will have the same direction. This is similar to burden tests that have been widely used for rare variant analysis^22^ where a single parameter can accumulate evidence for association without increasing the number of degrees of freedom. When interaction effects are null on average (i.e. if interaction effects are both negative and positive so that the sum of interaction coefficients tend to zero), the single SNP approach will generally outperform the risk score-based approach. Conversely, if interaction effects tend to be in the same direction, the risk score-based approach can have dramatically higher power.^14^

## Methods

### Study sample

The present analysis relies on the Hancock *et al.*^9^ genome-wide meta-analysis for main genetic effects plus interaction effects with smoking in relation to pulmonary function among 50 047 participants (56% women) of European ancestry from 19 studies. The mean age was 53 years at the time of pulmonary function testing. Approximately 15% were current smokers and 56% were ever smokers. Among ever smokers, the average pack-years of smoking was 21. Supplementary Table 3 (available as Supplementary data at *IJE* online) provides the main characteristics of the studies included; complete details of study-specific pulmonary function testing protocols have been published.^4^ For studies with spirometry at a single visit, we analysed FEV_1_/FVC and FEV_1_ measured at that visit. For studies with spirometry at more than one visit, measurements from the baseline visit or the most recent examination with spirometry data was used. Smoking history (current, former and never smoking) was ascertained by questionnaire at the time of pulmonary function testing. Pack-years of smoking were calculated for current and past smokers by multiplying smoking amount (packs per day) and duration (years smoked). Approximately 2.5 million autosomal SNPs were tested for interaction with smoking status (ever smoking vs never smoking) and pack-years, for two outcomes: FEV**_1_** and FEV**_1_**/FVC (see next section). We also used two independent datasets of individuals of European ancestry to test for replication. The first replication dataset included 8859 unrelated individuals, and the second dataset included 9457 family-based individuals. The look-up was done in the GWAS for marginal genetic effects done separately in ever and never smoker as part of a recent meta-analysis of FEV_1_ and FEV_1_/FVC.^23^

### Single SNP-by-smoking interaction

The analysis performed in this study used summary statistics data from the aforementioned meta-analysis of 19 studies performed by Hancock et al.^9^ In brief, each of the 19 studies derived the residuals of FEV**_1_** and FEV**_1_**/FVC after regressing out age, age^2^, sex, standing height, principal component eigenvectors of genotypes and recruitment site if applicable. The residuals were normalized using a rank-based inverse normal transformation. Single SNP interaction effects were assessed using the following model (see Supplementary Note, available as Supplementary data at *IJE* online):

(1) $$Y∼\beta_{0}+\beta_{G}G+\beta_{GE_{k}}GE_{k}+\sum_{l=1\ldots 3}\beta_{E_{l}}E_{l}\text{ ,}$$

where *β_G_* and $\beta_{E_{l}}$ are the main effect of the SNP *G* and exposure *E_l_*, $\beta_{GE_{k}}$ is the interaction effect between *G* and exposure *E_k_*, and *β_0_* the intercept.

Detailed description of studies used in the replication analysis can be found in Soler Artigas *et al.*^23^ In brief, linear regression of age, age^2^, sex, height and principal components for population structure was undertaken on FEV_1_ and FEV_1_/FVC separately for ever smokers and never smokers. The residuals were normalized using a rank-based inverse normal transformation, again separately in ever smokers and never smokers. These transformed residuals were then used as the phenotype for association testing under an additive genetic model in each exposure strata. Inference of the interaction effects from the exposure-stratified analyses are described in the Supplementary Note (available as Supplementary data at *IJE* online).

### Multivariate interaction analysis overview

First, we considered an unweighted genetic risk score-by-smoking interaction where the risk score simply sums the number of risk alleles (i.e. alleles associated with a lower pulmonary function). This unweighted genetic risk score is most powerful when the interaction effects have the same direction as marginal SNP effects (i.e. the harmful effects of smoking are magnified in individuals with a genetic predisposition to reduced pulmonary function). Second, we used a weighted genetic risk score where SNPs were weighted by the absolute value of their marginal effect estimates obtained from stage 1 screening of FEV**_1_** and FEV**_1_**/FVC from Soler Artigas *et al.*^4^ (Supplementary Table 1, available as Supplementary data at *IJE* online). This weighting scheme is most powerful when the magnitude of interaction effects is proportional to the SNP marginal effects. Finally, for our third multivariate analysis, we derived a standard omnibus test of all interaction effects. This test will retain power in the presence of effects in both directions or of different magnitudes. Although there is strong correlation among the 12 tests performed (these three models, considering interaction with two smoking metrics, ever/never smoking or pack-years, for the two pulmonary function metrics FEV1 and FEV1/FVC), we used a stringent Bonferroni *P*-value correction threshold of 4 × 10^–3^ to account for multiple testing.

When raw data are available, the weighted genetic risk score (*GRS*) is usually expressed as *GRS* = Σ*_m_*[*w_i_* × *G_i_*], where *m* is the number of SNPs included in the genetic risk score and *w* = (*w*_1_,..*w_m_*) are the weights attributed to each single SNP. Following previous notation, the test of interaction between the GRS and the exposure *E_k_* can be applied using the following model:

(2) $$Y∼\gamma_{0}+\gamma_{GRS}\times GRS+\gamma_{INT}\times GRS\times E_{k}+\sum_{l=1\ldots 3}{\text{γ}}_{E_{l}}\times E_{l}\text{ ,}$$

where *γ*_0_, *γ_GRS_*, $\gamma_{E_{l}}$ and *γ_INT_* are the intercept, the main effect of the GRS, the main effect of the exposure *E_l_* and the interaction effect between *E_k_* and the GRS, respectively. However, because individual-level data were not directly available, we performed the test of *γ_INT_* from summary statistics of interaction effects using an inverse-variance weighted sum as proposed by Aschard.^14^ The chi-square for the interaction term *γ_INT_* was derived as follows:

(3) $$\chi_{int}^{2}=\frac{{\left(\sum_{i=1\ldots m}\frac{w_{i}\times {\hat{\beta }}_{G_{i}E_{k}}}{{\hat{\sigma }}_{\beta_{G_{i}E_{k}}}^{2}}\right)}^{2}}{\sum_{i=1\ldots m}\frac{w_{i}^{2}}{{\hat{\sigma }}_{\beta_{G_{i}E_{k}}}^{2}}},$$

where ${\hat{\beta }}_{G_{i}\times E_{k}}$ and ${\hat{\sigma }}_{\beta_{G_{i}\times E_{k}}}^{2}$ are the estimated effects and variance of the interaction between the exposure *E_k_* and the SNP *G_i_* obtained from Equation (1) and *w_i_* is the weight applied to SNP *G_i_.* Under the null hypothesis of no interaction effect, $\chi_{int}^{2}$ follows a chi-squared distribution with one degree of freedom.

The standard omnibus test of all interaction effects consisted of evaluating jointly $\alpha_{G\times E_{k}}=(\alpha_{G_{1}\times E_{k}},\ldots ,\alpha_{G_{m}\times E_{k}})$ from the model:

(4) $$Y∼\alpha_{0}+\sum_{i=1\ldots m}\left[\alpha_{G_{i}}\times G_{i}\right]+\sum_{i=1\ldots m}\left[\alpha_{G_{i}\times E_{k}}\times G_{i}\times E_{k}\right]+\sum_{l=1\ldots 3}\alpha_{E_{l}}\times E_{l},$$

where $\alpha_{0}$, $\alpha_{G_{i}}$, $\alpha_{E_{l}}$ and $\alpha_{G_{i}\times E_{k}}$ are the intercept, the main effects of SNP *G_i_* and the exposure *E_l_*, and the interaction effect between *G_i_* and *E_k_.* Leveraging the independence between the SNPs considered (a single SNP was selected for each independent locus), we also derived the omnibus test using summary statistics. Under this independence assumption, the *G_i_* × *E_k_* interaction terms would also be independents,^14^ so that it can be performed by summing the chi-square from each univariate interaction test to form a chi-square with *m* degrees of freedom as follows:

(5) $$\chi_{\mathrm{omnibus}}^{2}=\sum_{i=1\ldots m}\frac{{\hat{\beta }}_{G_{i}\times E_{k}}^{2}}{{\hat{\sigma }}_{\beta_{G_{i}\times E_{k}}}^{2}},$$

where ${\hat{\beta }}_{G_{i}\times E_{k}}$ and ${\hat{\sigma }}_{\beta_{G_{i}\times E_{k}}}^{2}$ are the estimated effects and variance of the interaction between the exposure *E_k_* and the SNP *G_i_* obtained from Equation (1).

### Relative risk in ever smokers vs never smokers

GRS interaction effects can further be translated in terms of risk prediction. For pulmonary function, low FEV**_1_** or FEV**_1_**/FVC increases the risk of death^24^ and together they form the basis for the diagnosis of COPD.^1–3^ COPD stage 2 or higher are defined by the Global Initiative for Chronic Obstructive Lung Disease (GOLD) as FEV_1_/FVC < 0.70 and FEV**_1_** < 80% of the predicted value. According to recent studies,^2^^,^^25^ between 5% and 20% of European ancestry adults are expected to have FEV**_1_**/FVC < 0.70, depending on smoking characteristics and age distribution. Several studies argue for a more stringent threshold to define COPD^25^^,^^26^ based on lower limit of normal predicted value, rather than a fixed absolute value, to prevent disease misclassification.

To explore the impact of interaction effect on the risk of disease, we derived the relative risk (RR) of having FEV**_1_**/FVC below a given threshold (1%, 5% and 20%) in ever smokers vs never smokers conditional on the unweighted GRS. This quantity is defined as the joint probability of having both FEV**_1_**/FVC in the interval [–∞, FEV_1_/FVC*_up_*] and the GRS in the interval [*GRS_low_*,*GRS_up_*]. This can be expressed as the following integral:

(6) $$\overset{{\text{FEV}}_{1}/{\text{FVC}}_{up}}{\underset{-\infty }{\int }}\overset{GRS_{up}}{\underset{GRS_{low}}{\int }}f_{1}\left(y\text{|}g,e\right)\times f_{2}\left(g\text{|}e\right)dydg,$$

where *y*, *e* and *g* are FEV**_1_**/FVC, smoking status and the GRS, respectively, and *f*_1_ and *f*_2_ are the probability density function of y and *g.* The detailed derivation of the above integral is available as Supplementary data at *IJE* online.

## Results

We selected 26 loci previously found to be associated with FEV**_1_** or FEV**_1_**/FVC at genome-wide significance (*P* < 5 × 10^–8^) in marginal association tests^4^^,^^11^^,^^27^ (i.e. not including interaction effects with smoking exposures) and replicated in the GWAS by Soler Artigas *et al.*,^4^ the largest meta-analysis of marginal genetic effect conducted for these two traits in the general population. Additional loci for these two phenotypes have been identified in two recent studies.^28^^,^^29^ However, these new loci were not included in our analysis because both these studies used a large cohort ascertained through smoking status. For each of the 26 selected loci, we choose the SNP with the strongest evidence for association (i.e. smallest *P*-value) with each of these phenotypes. The final list included 26 SNPs per phenotype, with only two SNPs being different between FEV**_1_** and FEV**_1_**/FVC as previously reported^4^ (Supplementary Table 1, available as Supplementary data at *IJE* online). Estimated interaction effects of these SNPs were extracted from the meta-analysis summary statistics for the four tests performed in the Hancock *et al.*^9^ analysis: SNP-by-smoking status (ever smoking vs never smoking) interaction effect on FEV**_1_** and FEV**_1_**/FVC; and SNP-by-smoking pack-years interaction effect on FEV**_1_**/FVC and FEV**_1_**. As shown in Supplementary Table 2 (available as Supplementary data at *IJE* online), nine SNPs showed nominal significance (*P* < 0.05) out of the 104 tests performed; however, none remained significant after accounting for multiple testing (Bonferroni corrected *P*-value threshold of 5 × 10^–4^). The minimum *P*-value was observed for the interaction between rs993925, near the TGFβ2 gene, and smoking status on FEV**_1_** [*β_int_* = –0.036, 95% confidence interval (CI), –0.009 to –0.032, *P* = 0.007].

Next, using these data, we conducted three multivariate (as opposed to single SNP) interaction analyses, testing jointly for the interaction effects between those SNPs and either smoking status or pack-years on the two phenotypes (FEV**_1_** and FEV**_1_**/FVC) for a total of 12 tests. As shown in Table 1, none of the multivariate interaction tests with pack-years was significant. However, four of the six multivariate interaction tests with smoking status (ever vs never) showed nominal significance, and two tests for FEV**_1_**/FVC had a *P*-value below the Bonferroni significance level (12 tests, P < 4 × 10^–3^). The strongest signal was observed for the unweighted genetic risk score-by-smoking status interaction effect on FEV**_1_**/FVC (*β_int_* = –0.036, 95% CI –0.040 to –0.032, *P* = 0.00057). The Cochran’s Q test for heterogeneity of the interaction effect across studies was not significant (*P* = 0.97) and the forest plot of study-specific results did not display any obvious outlier (Supplementary Figure 1, available as Supplementary data at *IJE* online).

**Table 1.** Multivariate interaction tests of the 26 loci associated with pulmonary function

Outcome	Exposure	Test	*^β_int_*	(CI)	*P*-value
FEV_1_	Smoking status^a^	*uGRS*	–0.0055	*(–0.011, 2.7 × 10^–5^)*	0.051
		*wGRS*	–0.21	*(–0.40, –0.033)*	**0.020**
		*CHISQ*	–	*–*	0.49
FEV_1_	Pack-years	*uGRS*	–1.6 × 10^–5^	*(–4.6* × *10^–5^, 1.4* × *10^–5^)*	0.30
		*wGRS*	–6.5 × 10^–4^	*(–1.6* × *10^–3^, 3.3* × *10^–4^)*	0.19
		*CHISQ*	–	*–*	0.46
FEV_1_/FVC	Smoking status	*uGRS*	–0.0099	*(–0.016, –0.0043)*	**0.00057** ^b^
		*wGRS*	–0.21	*(–0.33, –0.073)*	**0.0022** ^b^
		*CHISQ*	–	*–*	**0.026**
FEV_1_/FVC	Pack-years	*uGRS*	–4.4e-06	*(–3.6* × *10^–5^, 2.7* × *10^–5^)*	0.78
		*wGRS*	–6.5 × 10^–5^	*(–8.0* × *10^–4^, 6.6* × *10^–4^)*	0.85
		CHISQ	–	–	0.53

uGRS is the genetic risk score using equal weights to all SNPs; wGRS is the genetic risk score weighted by effect estimates from the marginal screening; CHISQ is the omnibus test of all interaction effects; *^β_int_* is the estimated interaction effect between the GRS and the outcome; and CI is the confidence interval of that estimate. Nominally significant tests are indicated in bold. ^a^Smoking status is defined as never smokers vs ever smokers. ^b^Significant *P*-value after Bonferroni correction.

**Figure 1.**
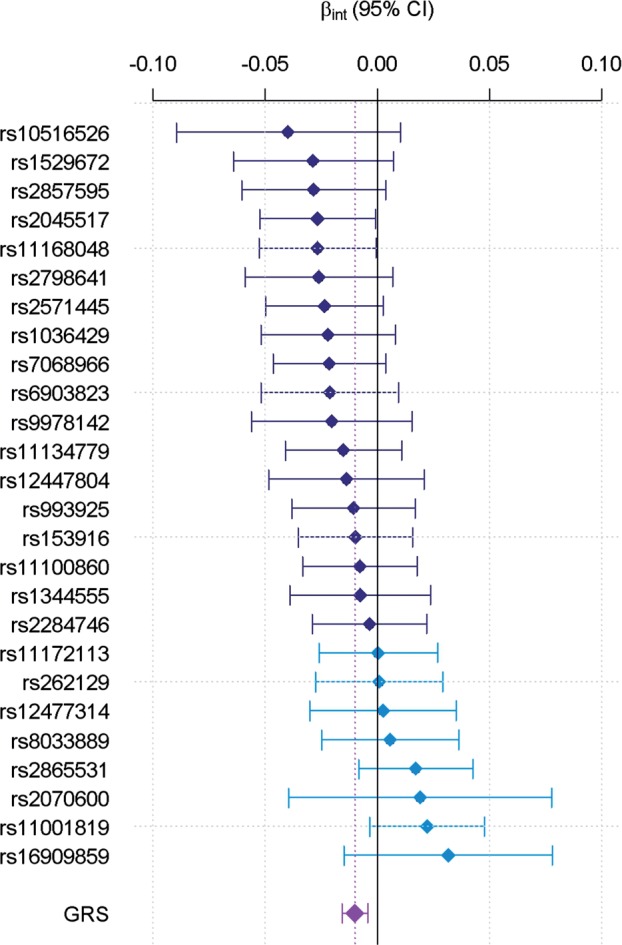
Distribution of interaction effects on FEV_1_/FVC. Single SNP risk allele-by-smoking status (ever/never) interaction effect estimates (*β_int_*) and 95% confidence intervals are plotted by increasing values. The unweighted genetic risk score-by-smoking status interaction is plotted at the bottom.

**Figure 2.**
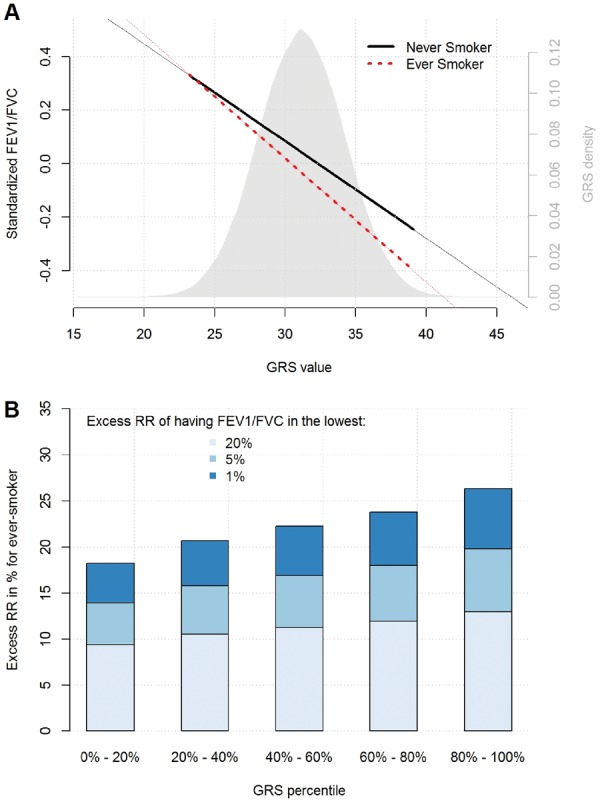
Overview of the unweighted genetic risk score-by-smoking interaction effect on FEV_1_/FVC. Upper panel (A) presents the distribution of the unweighted genetic risk score (GRS, grey density plot) and the relationship between the unweighted GRS and standardized FEV**_1_**/FVC in ever smokers (dashed line) and never smokers (solid line). Lower panel (B) shows the excess relative risk (RR) of having FEV**_1_**/FVC in the lowest 1%, 5% and 20% of the population for ever smokers compared with never smokers, as stratified by GRS quintiles.

The contrast between this significant risk score interaction and the absence of strong single SNP interaction effects can be explained by looking at the distribution of the single SNP interaction effect estimates. Figure 1 shows this distribution for the alleles associated with decreased FEV**_1_**/FVC. It highlights that, although the 95% CI of most single SNP interaction effects encompass the null (and therefore the absence of significant single SNP interaction effect), there is an enrichment for negative interaction effects. Indeed, even a binomial test can be used to confirm the unbalanced direction of interaction effects (18 of 26 interactions are negative leading to a *P*-value of 0.014 for a binomial test with an expected equiprobable distribution of 0.5). The genetic risk score-based interaction test exploits such enrichment by testing for the average interaction effect across all SNPs.^14^ As with any multivariate approach based on a composite null hypothesis, this result indicates that at least a subset of these 26 SNPs interact with smoking status, but does not allow us to determine which or how many SNPs are driving the genetic risk score-by-smoking interaction. The three other sets of single SNP interaction tests showed a similar (but not significant after correction for multiple testing) trend with enrichment for negative interactions (Supplementary Figures 2–4, available as Supplementary data at *IJE* online). We summarized the contribution of the unweighted genetic risk score-by-smoking interaction on FEV**_1_**/FVC in Table 2 and Figure 2A. This indicates that the deleterious effect of smoking is enhanced among carriers of the risk alleles or equivalently that the deleterious effect of smoking is reduced among subjects carrying the protective alleles.

**Table 2.** Summary of effect estimates for genetic risk score-by-smoking status interaction on FEV_1_/FVC

Predictors	Beta	SD	*P*-value
**From the marginal exposure model**			
Pack-years	–0.0030	0.00017	1.2 × 10^–71^
Current smoking	–0.040	0.0047	7.7 × 10^–18^
Smoking status^a^	–0.0023	0.0046	0.61
**From the interaction model**			
GRS	–0.0363	0.0021	3.9 × 10^–64^
GRS × Smoking status^a^	–0.0099	0.0029	5.7 × 10^–4^

GRS is the unweighted genetic risk score; beta is the effect estimates of each predictor; and SD the standard deviation of the each beta. ^a^Smoking status was defined as never smokers vs ever smokers.

We used two independent datasets, one of 8859 unrelated individuals and another of 9457 related individuals, to test for independent replication of our results (Supplementary Note, available as Supplementary data at *IJE* online). Although the interaction effects were not significant, both replication samples showed consistent negative GRS-by-ever smoking interaction effect on FEV1/FVC (${\hat{\beta }}_{int}$_ _= –0.0025, 95% CI –0.0165, 0.0115, *P* = 0.72 and ${\hat{\beta }}_{int}$_ _= –0.0030, 95% CI –0.0214, 0.0154, *P* = 0.74, and overall interaction effect in the combined replication datasets ${\hat{\beta }}_{int}$_ _= –0.0027, 95% CI –0.0136, 0.0082 *P* = 0.63) and a Cochran’s Q test for heterogeneity showed no significant difference in the three effect estimates (*P* = 0.51).

To quantify the impact of this result from a public health perspective, we estimated the impact of the genetic risk score-by-smoking interaction on having FEV**_1_**/FVC below 1%, 5% and 20% in the lower tails of the distribution in the population. Specifically, we derived the RR of having FEV**_1_**/FVC below these cut-off points (1%, 5% and 20%) in ever smokers compared with never smokers. Figure 2B quantifies the excess RR (i.e. the RR minus one) of individuals across five GRS quintiles. It highlights the higher risk associated with smoking among individuals carrying risk alleles (i.e. alleles associated with poorer pulmonary function) as compared with individuals carrying protective alleles (i.e. alleles associated with better pulmonary function). For example, among individuals with a GRS above the 80th percentile, smokers have on average a 26% excess RR of having FEV**_1_**/FVC in the lowest 1% of the population distribution, whereas ever smokers with a GRS below the 20th percentile have on average an 18% excess RR of falling in that same FEV**_1_**/FVC category compared with never smokers. Applying the same approach for FEV_1_, we observed a similar pattern (Supplementary Figure 5, available as Supplementary data at *IJE* online). However, as expected, the lower magnitude of the genetic risk score-by-ever smoking interaction on FEV_1_ implied a lower difference in RR between ever smokers and never smokers.

## Discussion

Using the largest dataset to date of European ancestry participants from the general population with pulmonary function (FEV_1_/FVC and FEV_1_), smoking and genetic data, we identified a gene-by-smoking interaction effect on FEV_1_/FVC by using a GRS composed of 26 SNPs identified and replicated in a prior GWAS meta-analysis of marginal genetic effects. To our knowledge, our study is the first to report a synergistic action of genes and smoking on pulmonary function (i.e. the reduction in FEV**_1_**/FVC as a result of smoking is greater among individuals who are genetically predisposed to lower FEV**_1_**/FVC ratio). Our study also highlights the importance of developing and applying alternative strategies to evaluate interaction effects for lung phenotypes along with other complex traits and diseases. The genetic risk score-based approach enabled us to identify an interaction when the standard univariate test (i.e. evaluating each single genetic variant for interaction independently) failed to identify any interactions.

Replication studies showed interaction effect estimates in the same direction as the discovery study but were not significant, and the magnitude of interaction effects were substantially smaller. We acknowledge that, despite careful evaluation of the interaction effects in the discovery sample, the observed signal might be overestimated or confounded by unmeasured complex factors. However, we can a priori rule out a systematic bias of the single SNP interaction effects in the discovery study, because the genomic inflation factor λ, defined as the ratio of the median of the empirically observed distribution of the test statistic to the expected median,^30^ was not substantially different from 1 (λ = 1.044 for FEV1/FVC and smoking status). Instead, differences in significance and effect estimates might be partly explained by the limited sample size in the replication study and differences in the analytical design. Indeed, the discovery analysis was performed using a saturated model including three smoking exposures and explicitly modelled the interaction effect. In comparison, the replication analysis was not adjusted for current smoking status and pack-year, and the interaction effect was approximated from analyses stratified by smoking status outcome, which has some limitations (see Supplementary Note and Supplementary Figure 6, available as Supplementary data at *IJE* online). Previous work has shown that combined analyses are more powerful when effects exist in both strata,^31^ as observed in discovery study. Further, even with *N* = 18 316 individuals in the combined replication population, we are underpowered. This sample size provides less than 50% power, at nominal significance of 5%, to detect interaction effects with the GRS.

Genetic risk score-by-exposure interaction can have higher clinical value than the identification of single SNP-by-exposure interaction by capturing a wealth of information in a single measure to identify subgroups in the population whose genetic background makes them more susceptible to the deleterious effects of smoking.^19^^,^^32^^,^^33^ Indeed, if single SNP-by-smoking interactions are distributed unconditionally on the marginal genetic effect (i.e. interaction effects are equally likely to be positive or negative given that the coded alleles are the risk alleles), the genetic effect is expected to be similar between ever and never smokers. The enrichment for negative interactions we identified through our GRS approach reveals a stronger genetic component among the ever smoker subgroup in the population and can allow the implementation of more efficient implementation of prevention strategies. For example, in the public health setting, programmes targeting smoking cessation campaigns to individuals who are genetically predisposed to low pulmonary function may have a stronger impact in preventing COPD.

**Figure 3.**
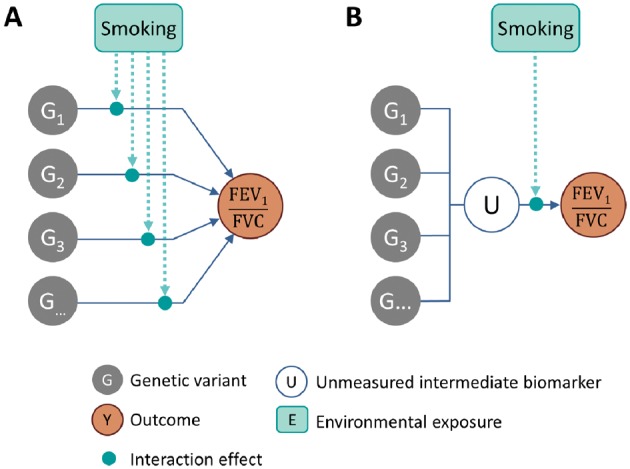
Underlying causal model. Potential causal diagrams underlying the gene and smoking interaction effects on FEV_1_/FVC. Panel (A) presents a scenario where each genetic variant influences the outcome through a SNP-specific pathway, and interactions with the environmental exposure take place along these pathways. Panel (B) presents an alternative (and simpler) model where multiple genetic variants influence an unmeasured intermediate biomarker U, which effect on FEV_1_/FVC depends on smoking. In scenario (A), the single SNP-by-smoking interaction test is the optimal approach, whereas, in scenario (B), the single SNP-by-smoking interaction test can become inefficient, and interaction would be easier to detect using a genetic risk score-by-smoking interaction test, because it summarizes all interaction effects in a single test.

Our results may also elucidate biological mechanisms underlying the interplay between genes and smoking in pulmonary function. In particular, the higher statistical power for the genetic risk score-based interaction test points towards the potential presence of an unmeasured intermediate biomarker mediating the effect of the 26 loci on FEV_1_/FVC. As shown in Figure 3, the most parsimonious model (i.e. the less complex following Occam’s razor) that would explain multiple interactions going in the same direction (Figure 1) implies that the genetic variants together influence an intermediate biomarker, which itself interacts with smoking. Future studies with extended genomic data, including transcriptomic, proteomic or metabolomic data, might be able to further assess such an hypothesis by evaluating (i) the effect of the GRS on those biomarkers and (ii) testing for interactions between smoking and the candidate biomarkers identified at step (i).

This study has some limitations. The 26 selected variants together explain a relatively small proportion of the additive genetic variance in FEV_1_/FVC and in FEV_1_.^4^ However, GWAS with increasing sample sizes will likely continue to provide additional associated genetic variants to further assess the role of SNP-by-smoking interaction effects on pulmonary phenotypes and may increase the gap between smokers and never smokers to allow for a significant impact in the clinic or at the population level. Moreover, we focused on genetic variants previously found to be associated at genome-wide significance level, but future studies might consider less stringent criteria to select genetic variants, including those with only suggestive evidence, or alternatively candidate variants with functional annotation relevant to the outcomes and exposures in question. Obviously, the signal-to-noise ratio might decrease when relaxing the constraint on the SNP selection. However, as we recently showed, additional gain in statistical power might be achieved even if a substantial proportion of the variants do not interact with the exposure.^14^ Finally, investigation of interaction effects with other environmental exposures such as second-hand smoke, air pollution, asbestos or occupational risks may lead to a more comprehensive understanding of the biological and epidemiological significance of these variants.

In summary, the identification of interaction effects between genetic variants and environmental exposures in human traits is recognized as extremely challenging, and this quest has been mostly unsuccessful so far. In this study, we discovered novel gene-by-smoking interactions using risk scores that were not observed at the level of individual genetic variants. This risk score analysis suggests that persons with a greater genetic predisposition to low pulmonary function are more susceptible to the deleterious effects of smoking. By extension, the use of a GRS may help predict which smokers will fall below thresholds that establish the diagnosis of COPD.

## Supplementary Data

Supplementary data are available at *IJE* online.

## Supplementary Material

Supplementary Online Data Supplement
