# Supplementary material for: Evidence for large-scale gene-by-smoking interaction effects on pulmonary function
Source: Int J Epidemiol. 2017 Jan 12;46(3):894–904. doi: 10.1093/ije/dyw318 (PMC5837518; doi:10.1093/ije/dyw318)
Supplement: Supplementary Online Data Supplement [file supplementary_online_data_supplement_dyw318.pdf]

Evidence for Large-Scale Gene-by-Smoking Interaction Effects on Pulmonary Function

Hugues Aschard, Martin D. Tobin, Dana B. Hancock, David Skurnik, Akshay Sood, Alan James, Albert Vernon Smith, Ani W. Manichaikul, Archie Campbell, Bram P. Prins, Caroline Hayward, Daan W Loth, David J. Porteous, David P Strachan, Eleftheria Zeggini, George O'Connor, Guy G. Brusselle, H. Marike Boezen, Holger Schulz, Ian J. Deary, Ian P. Hall, Igor Rudan, Jaakko Kaprio, James F. Wilson, Jemma B. Wilk, Jennifer E. Huffman, Jing Hua Zhao, Kim de Jong, Leo-Pekka Lyytikäinen, Louise V. Wain, Marjo-Riitta Jarvelin, Mika Kähönen, Myriam Fornage, Ozren Polasek, Patricia A. Cassano, R. Graham Barr, Rajesh Rawal, Sarah E. Harris, Sina A. Gharib, Stefan Enroth, Susan R. Heckbert, Terho Lehtimäki, Ulf Gyllensten, Understanding Society Scientific Group, Victoria E. Jackson, Vilmundur Gudnason, Wenbo Tang, Josée Dupuis, María Soler Artigas, Amit D. Joshi, Stephanie J. London, Peter Kraft

Online Data Supplement

Single SNP-by-smoking interaction

Assuming that $E = (E_1, .. E_3)$ is a vector of smoking exposures including smoking status (ever/never), current smoking, and pack-years, E_k is the exposure tested for interaction (either smoking status or pack-years), and Y is the outcome (either FEV_1 or FEV_1/FVC), the parent study by Hancock et al.[9] they uses a model that saturate the main effect of smoking but only included a single interaction term:

$$Y \sim \beta_0 + \beta_G G + \beta_{GE_k} G E_k + \sum_{l=1...3} \beta_{E_l} E_l$$

where β_G and β_{E_l} are the main effect of G and exposure E_l, β_{GE_k} is the interaction effect between G and exposure E_k, and β_0 the intercept. For each single nucleotide polymorphisms (SNP), each outcome, and each interacting exposure E_k, Hancock et al.[1] used $\hat{\beta}_G$ and $\hat{\beta}_{GE_k}$, their variance $\hat{\sigma}_{\beta_G}$ and $\hat{\sigma}_{\beta_{GE_k}}$, and their covariance from *Equation 1* estimated within each study, and derived a meta-analysis joint test of β_G and β_{GE_k}. In this study, we used only the estimate of the interaction effect and its standard deviation ($\hat{\beta}_{GE_k}$ and $\hat{\sigma}_{\beta_{GE_k}}$), derived across all studies as part of the aforementioned meta-analysis to perform multivariate tests of interaction effects across multiple genetic variants. Finally, for clarity, main genetic effect refers to the estimated effect of genetic variants among never smokers, derived from a model with the interaction term. In contrast, marginal genetic effect refers to the estimated average genetic effect across all smoking categories, derived from a model without the interaction term.

Model characteristics

The main effect of smoking variables in the interaction model $\beta_{E_l}, l = (1, \dots 3)$ in *Equation 1* were not available in the summary statistics data. However their marginal effects, derived in a multivariate model similar to the one used in the genome-wide association (GWAS) but without the genetic component (no SNP main effect or SNP-by-smoking interaction effect), were available for each of the 19 studies.[1] For each of the three smoking exposures E, we first derived γ_{mE}, their marginal effect over all studies, using a standard inverse variance-weighted meta-analysis of study-specific estimates.

Using γ_{mE}, we then estimated $\gamma_{E_l}, l = (1, \dots 3)$, the main effect of smoking exposure from the genetic risk score (GRS)-by-smoking interaction model (*Equation 2*). For exposures $E_{l \neq k}$ not modeled to interact with the SNPs, the main effect was assumed to be equal to the marginal effect ($\gamma_{E_{l \neq k}} = \gamma_{mE_{l \neq k}}$). For E_k, the interacting exposure, the main effect estimate was approximated using the relationship defined in [2]:

$$\gamma_{E_k} = \gamma_{mE_k} - \gamma_{INT} \times \mu_{GRS}$$

where γ_{INT} is the interaction effect between the GRS and E_k, and μ_{GRS} is the mean of the GRS. The validity of this approximation mostly relies on independence between the GRS and E_k, but remains valid for low to moderate correlation (e.g., <0.1).

We then derived the mean and variance of each exposure across all studies using the sample size-weighted average. Study-specific descriptive statistics were available for all studies for ever/never smoking and pack-years, and for the largest studies for current smoking (Framingham Heart Study, Cardiovascular Health Study, Atherosclerosis Risk in Communities, LifeLines, European Prospective Investigation into Cancer and Nutrition, and British 1958 Birth Cohort). The means were used to approximate γ_0, the intercept of the interaction models with the GRS (*Equation 2*). Because both outcomes were standardized to have mean 0, γ_0 equals the opposite of the average effect of all predictors:

$$\gamma_0 = -\gamma_{GRS} \times \mu_{GRS} - \gamma_{INT} \times \mu_{GRS \times E_k} - \sum\nolimits_{l=1 \dots 3} \left(\gamma_{E_l} \times \mu_{E_l}\right)$$

where $\mu_{.}$ are the mean of the predictors considered.

Derivation of Relative risk in ever smokers against never-smokers

We aimed at estimating the joint probability of having both $\mathrm{FEV_1/FVC}$ in the interval $[-\infty, \mathrm{FEV_1/FVC}_{up}]$ and the GRS in the interval $[GRS_{low}, GRS_{up}]$, which can be expressed as the following integral:

$$\int_{-\infty}^{\mathrm{FEV_1/FVC}_{up}} \int_{GRS_{low}}^{GRS_{up}} f_1(y|g) \times f_2(g)\, dy\, dg$$

In practice, we derived the bivariate cumulative distribution function of the GRS and FEV_1/FVC independently for ever smokers and never smokers using the R function *pmvnorm* from R package *mvtnorm* and the estimated effects from the interaction model. We assumed a normal conditional distribution of $\frac{\mathrm{FEV_1}}{FVC}$, which was standardized in the original analysis (i.e., $\sigma^2_{\frac{\mathrm{FEV_1}}{FVC}} = 1$), so that $\frac{\mathrm{FEV_1}}{FVC} \sim \mathcal{N}(\gamma_0 + \gamma_{GRS} \times \mu_{GRS}, 1)$ in never smokers and $\frac{\mathrm{FEV_1}}{FVC} \sim \mathcal{N}(\gamma_0 + (\gamma_{GRS} + \gamma_{INT}) \times \mu_{GRS} + \gamma_{E_k} + \sum_l \left(\gamma_{E_l} \times \mu_{E_l} | E_k = 1\right), 1)$ in ever smokers, where E_k is the ever-never smoking variable, γ_{E_k} its effect as defined in *Equation 2,* and $\mu_.$ are the mean of the predictors considered. We assumed the GRS was independent of the smoking variable, so that its distribution simply equals $\mathcal{N}(\mu_{GRS}, \sigma_{GRS})$. The covariance term of the bivariate distributions was defined as the GRS effect specific to each group times the standard deviation of the GRS (i.e., $cov\left(GRS, \frac{\mathrm{FEV_1}}{FVC} \middle| non-smokers\right) = \gamma_{GRS} \times \sigma_{GRS}$, and $cov\left(GRS, \frac{\mathrm{FEV_1}}{FVC} \middle| ever-smokers\right) = (\gamma_{GRS} + \gamma_{INT}) \times \sigma_{GRS}$).

Replication study

Two replication datasets were used. The first dataset included 8,859 unrelated individuals recruited as part of three studies: Lothian Birth Cohort 1936 (LBC1936, n = 991), United Kingdom Household Longitudinal Study (UKHLS, n = 7,449), and Young Finish Study (YFS, n = 419). The second dataset of 9,457 family-based samples included the following: CROATIA-Split (n = 493); GS:SFHS (n = 8,093); and NSPHS (Northern Sweden Population Health Study, n = 871). All datasets already had GWAS results available for marginal genetic effects stratified by ever-never smoking status as part of a recent meta-analysis of FEV_1 and FEV_1/FVC.[3] Detailed description of individual studies can be found in Soler Artigas et al.[3] except for UKLHS, which is described in the next section of this supplement.

Assuming the following stratified models for each SNP G_i, where G_i is coded additively (0, 1, or 2 corresponding to the number of coded allele): $Y_N \sim \gamma_0 + \gamma_{G_{i.never}} \times G_{i.never} + \boldsymbol{\gamma}_{\mathbf{C}} \times \mathbf{C}$ in never smokers, and $Y_S \sim \gamma_0 + \gamma_{G_{i.ever}} \times G_{i.ever} + \boldsymbol{\gamma}_{\mathbf{C}} \times \mathbf{C}$ in ever smokers, where γ_0 is the intercept, $\gamma_{G_{i.never}}$ is the marginal genetic effect in never smokers and $\gamma_{G_{i.ever}}$ is the marginal genetic effect in ever smokers, and $\boldsymbol{\gamma}_{\mathbf{C}}$ is the effect of the covariates $\mathbf{C}$. Single-SNP interaction effect estimates ($\hat{\beta}_{INT_i}$) and standard error ($\hat{\sigma}_{\beta_{INT_i}}$) were approximated using the following equations:

$$\hat{\beta}_{INT_i} = \hat{\gamma}_{G_{i.ever}} - \hat{\gamma}_{G_{i.never}}$$

$$\hat{\sigma}_{\beta_{INT_i}} = \sqrt{\hat{\sigma}^2_{\gamma_{G_{i.ever}}} + \hat{\sigma}^2_{\gamma_{G_{i.never}}} - 2\,\rho\,\hat{\sigma}_{\gamma_{G_{i.ever}}} \hat{\sigma}_{\gamma_{G_{i.never}}}}$$

Where ρ is the Spearman rank correlation estimates between $\hat{\gamma}_{G_{i.ever}}$ and $\hat{\gamma}_{G_{i.never}}$ derived across all SNPs from the GWAS. However, for cohorts of unrelated individuals, we assumed $\rho = 0$, so that $\hat{\sigma}_{\beta_{INT_i}}$ simplifies to:

$$\hat{\sigma}_{\beta_{INT_i}} = \sqrt{\hat{\sigma}^2_{\gamma_{G_{i.ever}}} + \hat{\sigma}^2_{\gamma_{G_{i.never}}}}$$

We then performed a meta-analysis of each SNP G_i across K studies using standard inverse-variance formula, that is:

$$\hat{\beta}_{INT_i.META} = \frac{\sum_K \frac{\hat{\beta}_{INT_i}}{\hat{\sigma}^2_{\beta_{INT_i}}}}{\sum_K \frac{1}{\hat{\sigma}^2_{\beta_{INT_i}}}}$$

$$\hat{\sigma}^2_{INT_i.META} = \frac{1}{\sum_K \frac{1}{\hat{\sigma}^2_{\beta_{INT_i}}}}$$

GRS-by-ever smoking interaction was then derived using the approach described in the Method section.

Note that this approach has limitations and might lead to biased interaction effect estimates in the presence of covariates associated with both the exposure and the outcome. For illustration purposes we generated series of 1,000 simulations each including 10,000 individuals. For each series three outcomes were simulated as a function of a genotype G with minor allele frequency 0.3, a binary exposure E_1 with frequency 0.5, and two normally distributed continuous exposures $E_2$and E_3present in either unexposed only or exposed only (i.e., had value of zero in either exposure strata).

$$Y_1 = \beta_0 + \beta_G G + \beta_{E_1} E_1 + \beta_{GE_1} GE_1 + \varepsilon$$

$$Y_2 = \beta_0 + \beta_G G + \beta_{E_1} E_1 + \beta_{E_2} E_2 + \beta_{GE_1} GE_1 + \varepsilon$$

$$Y_3 = \beta_0 + \beta_G G + \beta_{E_1} E_1 + \beta_{E_3} E_3 + \beta_{GE_1} GE_1 + \varepsilon$$

We plotted in **Supplementary Figure S6** the point estimates and power for the standard interaction model applied to Y_1, and the stratified approach described above and applied to Y_2 and Y_3. Both of the later analyses display biased estimates with magnitude increasing with β_{E_2} and β_{E_3}, respectively.

UKHLS

The UKHLS, also known as Understanding Society (https://www.understandingsociety.ac.uk), is a longitudinal panel survey of 40,000 households (England, Scotland, Wales, and Northern Ireland) that are representative of the UK population. Beginning in 2009, participants are surveyed annually and contribute information relating to their socioeconomic circumstances, attitudes, and behaviors via a computer-assisted interview. The study includes phenotypic data for a representative sample of participants for a wide range of social and economic indicators and a biological sample collection encompassing biometric, physiological, biochemical, and hematological measurements and self-reported medical history and medication use. The UKHLS has been approved by the University of Essex Ethics Committee, and informed consent was obtained from every participant.

For a subset of individuals who took part in a nurse health assessment, blood samples were taken and genomic DNA extracted. Of these, 10,484 were genotyped at the Wellcome Trust Sanger Institute using the Illumina Infinium HumanCoreExome-12 v1.0BeadChip.

Lung function measures in samples from England and Wales were conducted with the NDD Easy On-PC spirometer (NDD Medical Technologies, Zurich, Switzerland). Participants were excluded in the following cases: pregnancy, having had abdominal or chest surgery in the past 3 weeks, admitted to the hospital with a heart complaint in the past 6 weeks, having had eye surgery in the past 4 weeks, or having a tracheostomy. Subjects were asked to perform up to eight blows that ideally lasted at least 6 seconds, uninterrupted by coughing, glottis closure, laughing, or leakage of air. Upon completion, the measurements were rated either acceptable or unacceptable by the NDD Easy On-PC software.

The study included 3,293 males (44.2%) and 4,509 (60.5%) ever smokers. Average age was 53.10 (SD=15.94), average FEV_1 (in liter) was 2.89 (SD=0.90), and average FEV_1/FVC was 0.753 (SD=0.09).

Acknowledgments

Acknowledgments for all studies included in the prior meta-analyses, from which we derived results for the current study, were outlined in Hancock et al.[4] or Soler Artigas et al.[3] Acknowledgment for the cohorts with authors represented in the current study: **Cohorts for Heart and Aging Research in Genomic Epidemiology (CHARGE):** Infrastructure for the CHARGE Consortium is supported in part by the National Heart, Lung, and Blood Institute (NHLBI) grant R01HL105756. **Atherosclerosis Risk in Communities Study (ARIC)**: ARIC is carried out as a collaborative study supported by NHLBI contracts (HHSN268201100005C, HHSN268201100006C, HHSN268201100007C, HHSN268201100008C, HHSN268201100009C, HHSN268201100010C, HHSN268201100011C, and HHSN268201100012C), R01HL087641, R01HL59367, and R01HL086694; National Human Genome Research Institute (NHGRI) contract U01HG004402; and National Institutes of Health (NIH) contract HHSN268200625226C. The authors thank the staff and participants of the ARIC study for their important contributions. Infrastructure was partly supported by grant number UL1RR025005, a component of the NIH and NIH Roadmap for Medical Research. **Cardiovascular Health Study (CHS):** CHS research was supported by NHLBI contracts HHSN268201200036C, HHSN268200800007C, N01HC55222, N01HC85079, N01HC85080, N01HC85081, N01HC85082, N01HC85083, and N01HC85086; and NHLBI grants U01HL080295, R01HL087652, R01HL105756, R01HL103612, and R01HL120393 with additional contribution from the National Institute of Neurological Disorders and Stroke. Additional support was provided through R01AG023629 from the National Institute on Aging. A full list of principal CHS investigators and institutions can be found at CHS-NHLBI.org. The provision of genotyping data was supported in part by the National Center for Advancing Translational Sciences, CTSI grant UL1TR000124, and the National Institute of Diabetes and Digestive and Kidney Diseases (NIDDK) Diabetes Research Center grant DK063491 to the Southern California Diabetes Endocrinology Research Center. The content is solely the responsibility of the authors and does not necessarily represent the official views of the National Institutes of Health. **Framingham Heart Study (FHS)**: FHS research was conducted in part using data and resources of the NHLBI and Boston University School of Medicine. This work was partially supported by NHLBI (contract no. N01-HC-25195 and HHSN268201500001I) and its contract with Affymetrix for genotyping services (contract no. N02-HL-6-4278). **Multi-Ethnic Study of Atherosclerosis (MESA):** The MESA Lung study and the MESA SHARe project are conducted and supported by NHLBI in collaboration with MESA investigators. Support for MESA is provided by contracts N01-HC-95159, N01-HC-95160, N01-HC-95161, N01-HC-95162, N01-HC-95163, N01-HC-95164, N01-HC-95165, N01-HC-95166, N01-HC-95167, N01-HC-95168, N01-HC-95169, UL1-TR-001079, UL1-TR-000040, and DK063491. The MESA Lung Study is funded by R01HL077612, RC1100543, and R01H093081. Funding for SHARe genotyping was provided by NHLBI Contract N02-HL-64278. Genotyping was performed at Affymetrix (Santa Clara, California, USA) and the Broad Institute of Harvard and MIT (Boston, Massachusetts, USA) using the Affymetrix Genome-Wide Human SNP Array 6.0. **British 1958 Birth Cohort (B58C)**: We acknowledge use of phenotype and genotype data from the B58C DNA collection, funded by the Medical Research Council grant G0000934 and the Wellcome Trust grant 068545/Z/02. Genotyping for the B58C-WTCCC subset was funded by the Wellcome Trust grant 076113/B/04/Z. The B58C-T1DGC genotyping used resources provided by the Type 1 Diabetes Genetics Consortium, a collaborative clinical study sponsored by the NIDDK, National Institute of Allergy and Infectious Diseases, NHGRI, National Institute

of Child Health and Human Development, and Juvenile Diabetes Research Foundation International and supported by U01 DK062418. B58C-T1DGC GWAS data were deposited by the Diabetes and Inflammation Laboratory, Cambridge Institute for Medical Research (CIMR), University of Cambridge, which is funded by Juvenile Diabetes Research Foundation International, the Wellcome Trust, and the National Institute for Health Research Cambridge Biomedical Research Centre; the CIMR is in receipt of a Wellcome Trust Strategic Award (079895). The B58C-GABRIEL genotyping was supported by a contract from the European Commission Framework Programme 6 (018996) and grants from the French Ministry of Research. **Northern Finland Birth Cohort 1966 (NFBC1966)**: NFBC1966 received financial support from the Academy of Finland (project grants 104781, 120315, 129269, 1114194, 24300796, Center of Excellence in Complex Disease Genetics and SALVE), University Hospital Oulu, Biocenter, University of Oulu, Finland (75617), NHLBI grant 5R01HL087679-02 through the STAMPEED program (1RL1MH083268-01), NIH/NIMH (5R01MH63706:02), ENGAGE project and grant agreement HEALTH-F4-2007-201413, EU FP7 EurHEALTHAgeing -277849, the Medical Research Council, UK (G0500539, G0600705, G1002319, PrevMetSyn/SALVE) and the MRC, Centenary Early Career Award. The program is currently being funded by the H2020-633595 DynaHEALTH action and academy of Finland EGEA-project (285547). The DNA extractions, sample quality controls, biobank upkeep, and aliquotting was performed in the National Public Health Institute, Biomedicum Helsinki, Finland, and supported financially by the Academy of Finland and Biocentrum Helsinki. We thank the late Professor Paula Rantakallio (launch of NFBCs), and Ms. Outi Tornwall and Ms. Minttu Jussila (DNA biobanking). The authors would like to acknowledge the contribution of the late Academian of Science Leena Peltonen. **Orkney Complex Disease Study (ORCADES)**: DNA extractions were performed at the Wellcome Trust Clinical Research Facility in Edinburgh. We would like to acknowledge the invaluable contributions of the research nurses in Orkney, the administrative team in Edinburgh and the people of Orkney.

HA was supported by R21HG007687. SJL was supported by the Intramural Research Program of the NIH, National Institute of Environmental Health Sciences. MDT, LVW. and MSA were supported by the National Institute for Health Research (NIHR). The views expressed are those of the authors and not necessarily those of the NHS, the NIHR, or the Department of Health. MDT holds a Medical Research Council Senior Clinical Fellowship (G0902313). PAC and DBH were supported by R21HL125574. JFW was supported by the Chief Scientist Office of the Scottish Government (CZB/4/276, CZB/4/710) and the EU FP6 (LSHG-CT-2006-018947). MRJ was supported by the National Public Health Institute, the Biomedicum Helsinki, Finland, the Academy of Finland, and the Biocentrum Helsinki. JHZ was supported by the Medical Research Council, the Cancer Research UK, the European Union, the Stroke Association, the British Heart Foundation, the Department of Health, the Food Standards Agency and the Wellcome Trust. JK was supported by the Academy of Finland (265240, 263278). IPH's research is funded by the MRC (G1000861).

The UKLHS is led by the Institute for Social and Economic Research at the University of Essex and funded by the Economic and Social Research Council. The survey was conducted by NatCen, and the genome-wide scan data were analyzed and deposited by the Wellcome Trust Sanger Institute. Information on how to access the data can be found on the Understanding Society website, https://www.understandingsociety.ac.uk/. The UKLHS includes authors Michaela Benzeval, Jonathan

Burton, Nicholas Buck, Annette Jäckle, Meena Kumari, Heather Laurie, Peter Lynn, Stephen Pudney, and Birgitta Rabe from the Institute for Social and Economic Research; and Dieter Wolke from University of Warwick.

Supplementary Table 1. Effect estimates from SNPs associated with cross-sectional FEV_1 or FEV_1/FVC measures.

chr	Gene	SNP	MAF[*]	A1	*FEV₁*			*FEV₁/FVC*			
					beta	*sd*	p	beta	*sd*	P	N
1	MFAP2	rs2284746	0.499	G	0.008	*0.007*	0.278	-0.042	*0.007*	$2.47x10^{-9}$	45944
1	TGFB2	rs993925	0.305	T	0.025	*0.007*	0.00151	0.04	*0.007*	$2.54x10^{-7}$	42402
2	HDAC4	rs12477314	0.201	T	0.032	*0.008*	0.000277	0.052	*0.008*	$4.48x10^{-9}$	45585
2	TNS1	rs2571445	0.396	G	0.047	*0.007*	$9.83x10^{-11}$	0.033	*0.007*	$4.46x10^{-6}$	45839
3	RARB	rs1529672	0.160	C	-0.037	*0.009*	0.000178	-0.06	*0.009*	$7.75x10^{-10}$	40624
3	MECOM	rs1344555	0.203	T	-0.042	*0.008*	$1.91x10^{-6}$	-0.019	*0.008*	0.0261	46067
4	FAM13A	rs2045517	0.400	T	-0.012	*0.007*	0.0893	-0.047	*0.007*	$2x10^{-11}$	47675
4	GSTCD-NPNT	rs10516526	0.066	G	0.108	*0.014*	$4.75x10^{-14}$	0.039	*0.014*	0.00617	47970
4	HHIP	rs11100860[a]	0.441	G (T)	0.047	*0.007*	$4.27x10^{-9}$	0.064	*0.007*	$6.81x10^{-20}$	47876
5	SPATA9	rs153916	0.454	T	-0.001	*0.007*	0.891	-0.033	*0.007*	$2.06x10^{-6}$	47530
5	ADAM19	rs11134779	0.359	G	-0.027	*0.007*	0.00024	-0.042	*0.007*	$6.01x10^{-9}$	48075
5	HTR4	rs11168048[b]	0.402	T (G)	-0.048	*0.007*	$2.43x10^{-10}$	-0.047	*0.007*	$5.97x10^{-11}$	44976
6	ZKSCAN3	rs6903823	0.206	G	-0.046	*0.008*	$2x10^{-7}$	-0.027	*0.008*	0.00228	47057
6	NCR3	rs2857595	0.160	G	0.04	*0.009*	$1.46x10^{-5}$	0.049	*0.009*	$7.86x10^{-8}$	45540
6	ARMC2	rs2798641	0.179	T	-0.046	*0.009*	$5.39x10^{-7}$	-0.047	*0.009*	$2.81x10^{-7}$	46369
6	AGER	rs2070600	0.050	T	0.025	*0.016*	0.127	0.126	*0.016*	$9.07x10^{-15}$	46314
6	LOC153910[c]	rs262129	0.294	G	0.031	*0.008*	$5.44x10^{-5}$	0.056	*0.008*	$2.91x10^{-13}$	47014
9	PTCH1	rs16909859	0.090	G	-0.014	*0.013*	0.293	0.08	*0.013*	$7.45x10^{-10}$	43353
10	CDC123	rs7068966	0.492	T	0.04	*0.007*	$1.19x10^{-8}$	0.045	*0.007*	$1.28x10^{-10}$	47085
10	C10orf11	rs11001819	0.470	G	-0.041	*0.007*	$1.42x10^{-8}$	-0.019	*0.007*	0.0065	45546
12	LRP1	rs11172113	0.396	T	-0.021	*0.007*	0.00355	-0.035	*0.007*	$1.36x10^{-6}$	45387
12	CCDC38	rs1036429	0.186	T	0.01	*0.008*	0.267	0.049	*0.008*	$1.24x10^{-8}$	47814
15	THSD4	rs8033889	0.202	T	-0.044	*0.009*	$3.01x10^{-7}$	-0.072	*0.008*	$2.03x10^{-17}$	46995
16	MMP15	rs12447804	0.195	T	-0.017	*0.009*	0.0802	-0.053	*0.009*	$7.12x10^{-8}$	35123
16	CFDP1	rs2865531	0.429	T	0.024	*0.007*	0.00063	0.039	*0.007*	$2.3x10^{-8}$	47594
21	KCNE2	rs9978142	0.144	T	-0.012	*0.009*	0.247	-0.048	*0.009*	$8.23x10^{-7}$	44577

Effect estimates and standard deviation of the 26 selected SNPs were extracted from stage 1 analysis of Soler Artigas et al.[3] We only included SNPs that were analyzed using at least 50% of the total sample at stage 1 (N>24,100). A1 is the coded allele.

[*] *From 1000Genomes European population.*

[a] *SNP rs1032296 was used instead of rs11100860 for FEV_1.*

[b] *SNP rs1985524 was used instead of rs11168048 for FEV_1.*

[c] *This locus is adjacent to the originally implicated GPR126 gene.*

Supplementary Table 2. Significance of univariate interaction effects for the 26 selected SNPs.

SNP ID	FEV_1				FEV_1/FVC			
	Smoking status		*Pack-year*		*Smoking status*		*Pack-year*	
	beta	*P-val*	*beta*	*P-val*	*beta*	*P-val*	*Beta*	*P-val*
rs2284746	0.005	0.66	$3.4x10^{-5}$	0.61	-0.003	0.78	$3.9x10^{-5}$	0.56
rs993925	-0.036	**0.0070**	$-1.9x10^{-5}$	0.80	-0.011	0.44	$-3.4x10^{-5}$	0.68
rs12477314	-0.004	0.81	$-9.0x10^{-6}$	0.92	0.003	0.87	$-1.9x10^{-4}$	**0.035**
rs2571445	-0.027	**0.040**	$4.1x10^{-5}$	0.56	-0.024	0.070	$1.1x10^{-5}$	0.88
rs1529672	-0.016	0.37	$7.5x10^{-5}$	0.40	-0.028	0.11	$2.1x10^{-4}$	**0.029**
rs1344555	-0.020	0.19	$7.5x10^{-5}$	0.36	-0.008	0.63	$-3.9x10^{-5}$	0.66
rs2045517	-0.008	0.54	$-9.2x10^{-5}$	0.18	-0.027	**0.039**	$-3.4x10^{-5}$	0.66
rs10516526	-0.035	0.16	$-2.2x10^{-4}$	0.12	-0.040	0.11	$-3.9x10^{-5}$	0.77
rs11100860[a]	0.008	0.53	$-1.2x10^{-5}$	0.87	-0.008	0.67	$-3.1x10^{-5}$	0.95
rs153916	-0.008	0.50	$1.1x10^{-4}$	0.090	-0.010	0.45	$-4.0x10^{-5}$	0.56
rs11134779	-0.012	0.36	$-4.6x10^{-5}$	0.51	-0.015	0.24	$6.0x10^{-5}$	0.42
rs11168048[b]	-0.010	0.44	$-2.4x10^{-5}$	0.72	-0.026	**0.028**	$-3.6x10^{-5}$	0.23
rs6903823	0.005	0.72	$4.1x10^{-5}$	0.59	-0.021	0.17	$4.5x10^{-5}$	0.58
rs2857595	-0.024	0.12	$-1.2x10^{-4}$	0.13	-0.028	0.080	$9.8x10^{-6}$	0.91
rs2798641	-0.002	0.89	$-1.7x10^{-4}$	**0.041**	-0.026	0.11	$-1.7x10^{-5}$	0.86
rs2070600	-0.022	0.46	$-1.1x10^{-4}$	0.39	0.019	0.51	$-7.0x10^{-5}$	0.62
rs262129	-0.001	0.97	$-8.6x10^{-5}$	0.25	0.001	0.95	$7.0x10^{-5}$	0.34
rs16909859	0.014	0.53	$2.2x10^{-6}$	0.99	0.032	0.17	$3.8x10^{-6}$	0.98
rs7068966	-0.005	0.66	$1.9x10^{-5}$	0.78	-0.021	0.090	$-1.6x10^{-4}$	**0.024**
rs11001819	0.001	0.94	$-3.3x10^{-5}$	0.62	0.022	0.080	$-2.0x10^{-5}$	0.77
rs11172113	0.011	0.41	$1.5x10^{-4}$	**0.033**	0.000	0.97	$-4.3x10^{-5}$	0.54
rs1036429	-0.005	0.72	$3.0x10^{-5}$	0.71	-0.022	0.14	$2.5x10^{-5}$	0.76
rs8033889	0.007	0.62	$-5.1x10^{-5}$	0.52	0.006	0.71	$-1.1x10^{-5}$	0.90
rs12447804	-0.005	0.78	$-5.2x10^{-4}$	0.28	-0.014	0.42	$-7.0x10^{-4}$	0.15
rs2865531	-0.011	0.39	$3.2x10^{-5}$	0.62	0.017	0.18	$9.3x10^{-5}$	0.19
rs9978142	0.017	0.33	$5.5x10^{-5}$	0.58	-0.020	0.26	$1.6x10^{-4}$	0.14

Nominally significant tests are indicated in bold. Betas were derived for the trait-decreasing alleles based on Table E1. SNPs are listed in order of chromosomal position as in Table S1.

[a]*SNP rs1032296 was used instead of rs11100860 for FEV_1.*

[b]*SNP rs1985524 was used instead of rs11168048 for FEV_1.*

Supplementary Table 3. Descriptive statistics of the 19 studies used in the initial screening.

Study (Country of origin)	Sample size *N (% female)*	Age (year) *Mean (SD)*	Height (cm) *Mean (SD)*	Never-smokers *N (%)*	Ever-smokers *N (%)*	Pack-years *Mean (SD)*	FEV$_1$ (mL) *Mean (SD)*	FVC (mL) *Mean (SD)*	FEV$_1$/FVC (%) *Mean (SD)*
AGES (Iceland)	1,696 *(59.4)*	76.2 *(5.6)*	166.7 *(9.4)*	813 *(47.9)*	883 *(52.1)*	24.5 *(21.9)*	2,128 *(690)*	2,865 *(848)*	73.9 *(10.5)*
ARIC (US)	8,934 *(52.7)*	54.3 *(5.7)*	168.8 *(9.4)*	3,620 *(40.5)*	5,314 *(59.5)*	28.9 *(21.6)*	2,943 *(744)*	3,993 *(980)*	73.7 *(7.9)*
B58C (UK)	4,605 *(50.3)*	44.5 *(0.4)*	169.2 *(9.3)*	1,376 *(29.9)*	3,229 *(70.1)*	15.7 *(12.1)*	3,288 *(757)*	4,164 *(980)*	79.5 *(8.1)*
CARDIA (US)	1,605 *(52.8)*	25.6 *(3.3)*	171.3 *(9.3)*	932 *(58.1)*	673 *(41.9)*	5.5 *(5.5)*	3,684 *(810)*	4,702 *(1,010)*	82.2 *(6.4)*
CHS (US)	3,140 *(61.0)*	72.3 *(5.4)*	164.6 *(9.4)*	1543 *(49.1)*	1597 *(50.9)*	33.2 *(26.9)*	2,116 *(659)*	3,005 *(866)*	70.5 *(10.5)*
ECRHS (EU)[1]	1,573 *(50.8)*	33.9 *(7.2)*	170.7 *(9.5)*	699 *(43.9)*	895 *(56.1)*	12.8 *(12.6)*	3,778 *(825)*	4,595 *(1029)*	82.6 *(6.6)*
EPIC obese cases (EU)[2]	1,084 *(57.8)*	59.1 *(8.8)*	165.93 *(9.24)*	489 *(44.3)*	595 *(54.9)*	18.2 *(14.1)*	2,355 *(694)*	2,839 *(872)*	83.8 *(10.2)*
EPIC population-based (EU)[2]	2,294 *(53.6)*	59.1 *(9.0)*	167.0 *(8.9)*	1,062 *(46.3)*	1,232 *(53.7)*	15.8 *(13.4)*	2,500 *(718)*	3,042 *(903)*	83.1 *(10.8)*
FHS (US)	7,694 *(53.9)*	51.9 *(14.6)*	168.5 *(9.7)*	3,556 *(46.2)*	4,138 *(53.8)*	22.8 *(21.5)*	3,038 *(944)*	4,025 *(1,144)*	75.1 *(8.0)*
Health ABC (US)	1,472 *(46.6)*	73.7 *(2.8)*	167.1 *(9.3)*	641 *(43.6)*	831 *(56.5)*	36.6 *(32.0)*	2,312 *(656)*	3,113 *(812)*	74.1 *(7.7)*
LifeLines (Netherlands)	2,616 *(59.9)*	54.2 *(9.5)*	173.0 *(9.1)*	981 *(37.7)*	1,621 *(52.3)*	14.5 *(12.6)*	3,172 *(804)*	4,233 *(1,007)*	75.0 *(7.5)*
MESA (US)	1,403 *(51.0)*	66.0 *(9.7)*	168.5 *(9.7)*	636 *(45.3)*	767 *(54.7)*	27.5 *(24.4)*	2,566 *(763)*	3,505 *(999.6)*	73.4 *(8.4)*
NFBC1966 (Finland)	3,564 *(50.5)*	31 *(0.0)*	171.5 *(9.3)*	1,648 *(46.2)*	1916 *(53.8)*	9.6 *(7.9)*	3,969 *(791)*	4,744 *(989)*	84.1 *(6.5)*
RS-I (Netherlands)	1,196 *(58.9)*	74.4 *(5.7)*	166.7 *(8.9)*	408 *(34.1)*	788 *(65.9)*	24.9 *(19.6)*	2,334 *(735)*	3,183 *(927)*	73.2 *(8.2)*
RS-II (Netherlands)	840 *(55.6)*	67.1 *(6.2)*	168.3 *(8.9)*	287 *(34.2)*	553 *(65.8)*	23.1 *(19.2)*	2,716 *(779)*	3,615 *(1,077)*	75.9 *(9.1)*
RS-III (Netherlands)	1,224 (56.8)	56.6 *(5.6)*	171.2 *(9.3)*	425 *(34.7)*	799 *(65.3)*	18.2 *(16.0)*	3,159 *(851)*	4,059 *(1,138)*	78.4 *(9.0)*
SAPALDIA Switzerland)	1,333 (52.6)	41.1 *(11.2)*	169.4 *(9.0)*	626 *(47.0)*	707 *(53.0)*	17.3 *(18.0)*	3,524 *(860)*	4,494 *(1,038)*	78.5 *(8.2)*
SHIP (Germany)	1,768 (51.2)	52.4 *(13.6)*	169.7 *(9.1)*	770 *(43.6)*	998 *(56.4)*	12.8 *(12.0)*	3,280 *(894)*	3,869 *(1,030)*	84.8 *(6.5)*
TwinsUK (UK)	2,006 (100)	54.2 *(14.1)*	161.8 *(6.4)*	1,242 *(61.9)*	764 *(38.1)*	13.7 *(21.4)*	2,599 *(606)*	3,251 *(650)*	79.7 *(7.7)*

AGES, Age, Gene/Environment Susceptibility; ARIC, Atherosclerosis Risk in Communities; B58C, British 1958 Cohort; CARDIA, Coronary Artery Risk Development in Young Adults; CHS, Cardiovascular Health Study; ECRHS, European Community Respiratory Health Survey; EPIC, European Prospective Investigation into Cancer and Nutrition; FEV$_1$, forced expiratory volume in the first second; FVC, forced vital capacity; FHS, Framingham Heart Study; Health ABC, Health, Aging, and Body Composition Study; MESA, Multi-Ethnic Study of Atherosclerosis; NFBC1966, Northern Finland Birth Cohort of 1966; RS, Rotterdam Study (cohorts I-III); SAPALDIA, Swiss Study on Air Pollution and Lung Diseases in Adults; SD, standard deviation; SHIP, Study of Health in Pomerania; SNP, single nucleotide polymorphism.

[1] The genetics data used in ECRHS include participants from 16 centers across 8 European countries (Estonia, France, Germany, Norway, Spain, Sweden, Switzerland, and UK).

[2] EPIC includes participants from 10 European countries: Denmark, France, Germany, Greece, Italy, The Netherlands, Norway, Spain, Sweden, and the United Kingdom.

Supplementary Figure 1. Forest plot of the GRS-by-ever smoking effect on FEV_1/FVC.

Effect estimate and 95% confidence interval are plotted for each of the 19 studies.

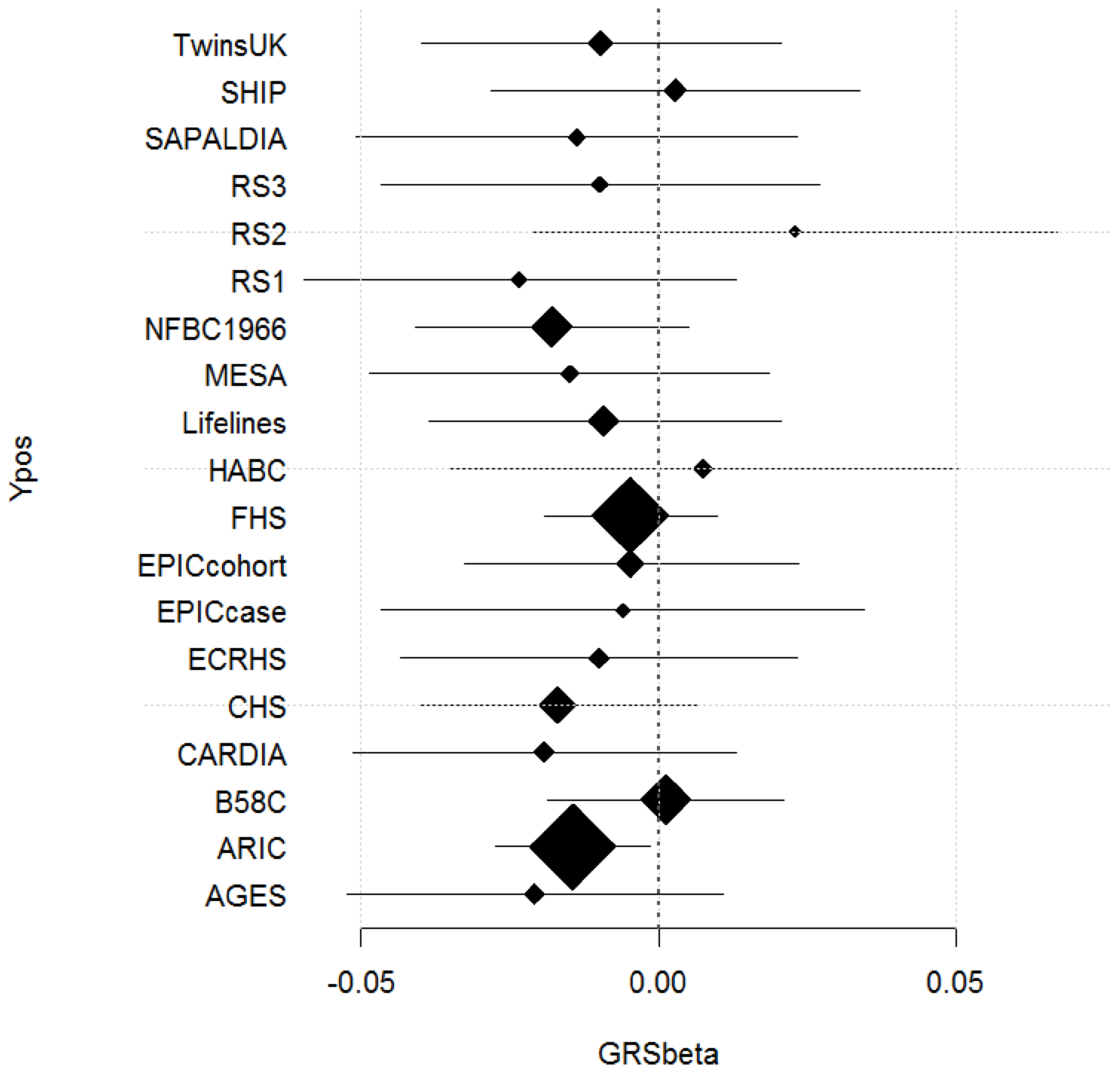

Supplementary Figure 2. Distribution of SNP-by-pack years interaction effects on FEV_1/FVC.

Single SNP risk allele-by-pack years interaction effect estimates (β_{int}) and 95% confidence intervals are plotted by increasing values. Negative and positive interactions are in dark blue and light blue, respectively. The unweighted GRS-by-pack years interaction is plotted in purple.

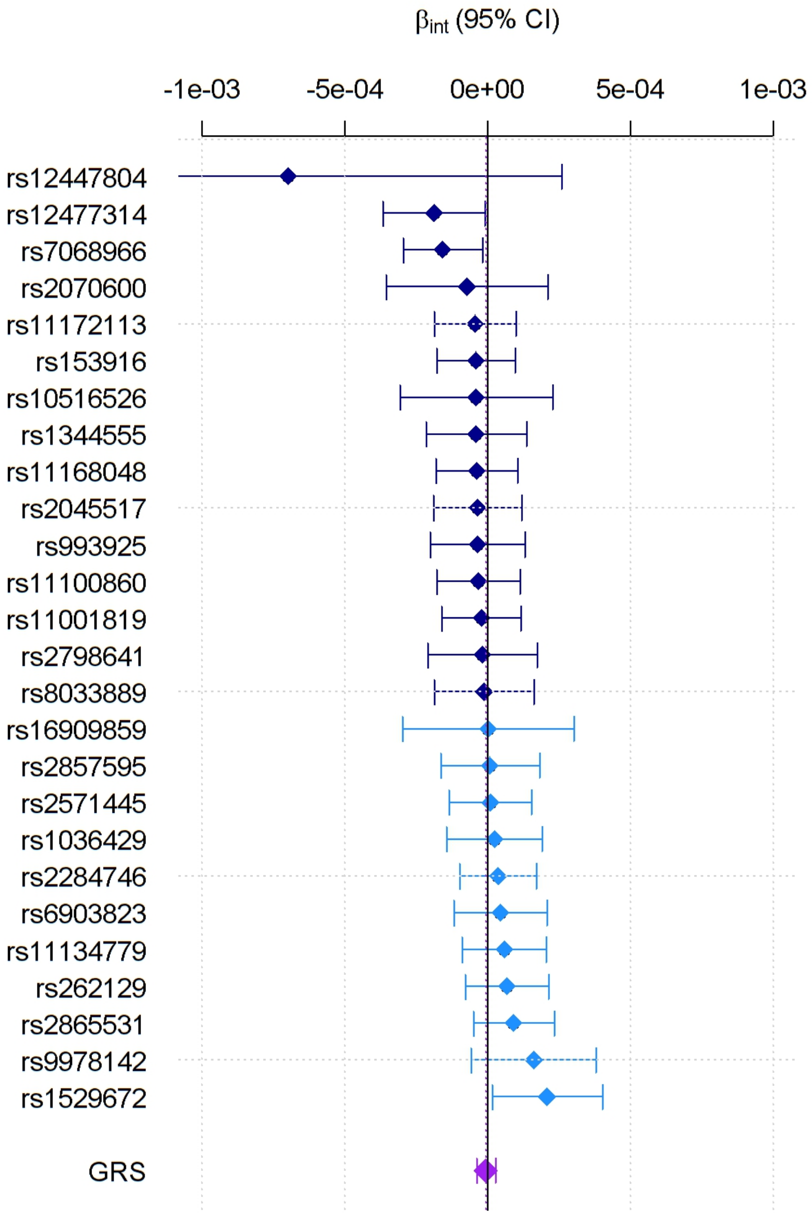

Supplementary Figure 3. Distribution of SNP-by-pack years interaction effects on FEV_1.

Single SNP risk allele-by-pack years interaction effect estimates (β_{int}) and 95% confidence intervals are plotted by increasing values. Negative and positive interactions are in dark blue and light blue, respectively. The unweighted GRS-by-pack years interaction is plotted in purple.

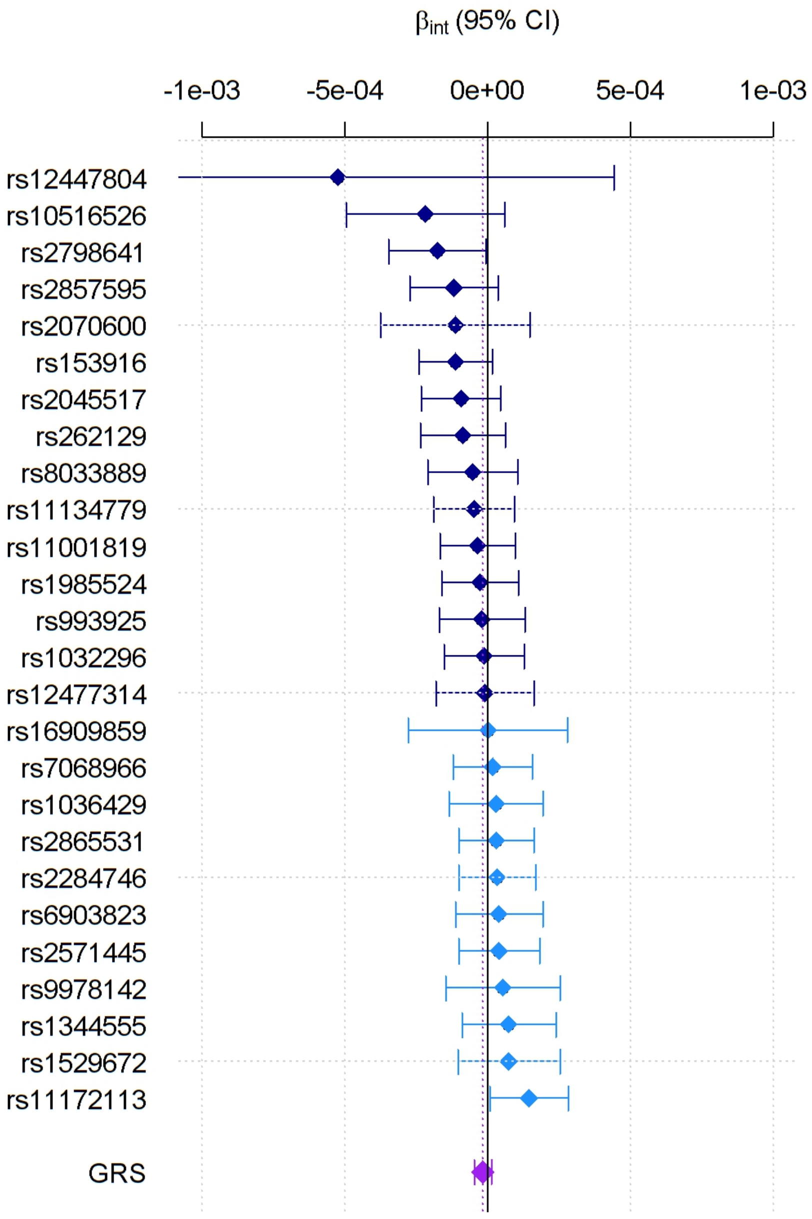

Supplementary Figure 4. Distribution of SNP-by-smoking status interaction effects on FEV_1.

Single SNP risk allele-by-smoking status (ever/never) interaction effect estimates (β_{int}) and 95% confidence intervals are plotted by increasing values. Negative and positive interactions are in dark blue and light blue, respectively. The unweighted GRS-by-smoking status interaction is plotted in purple.

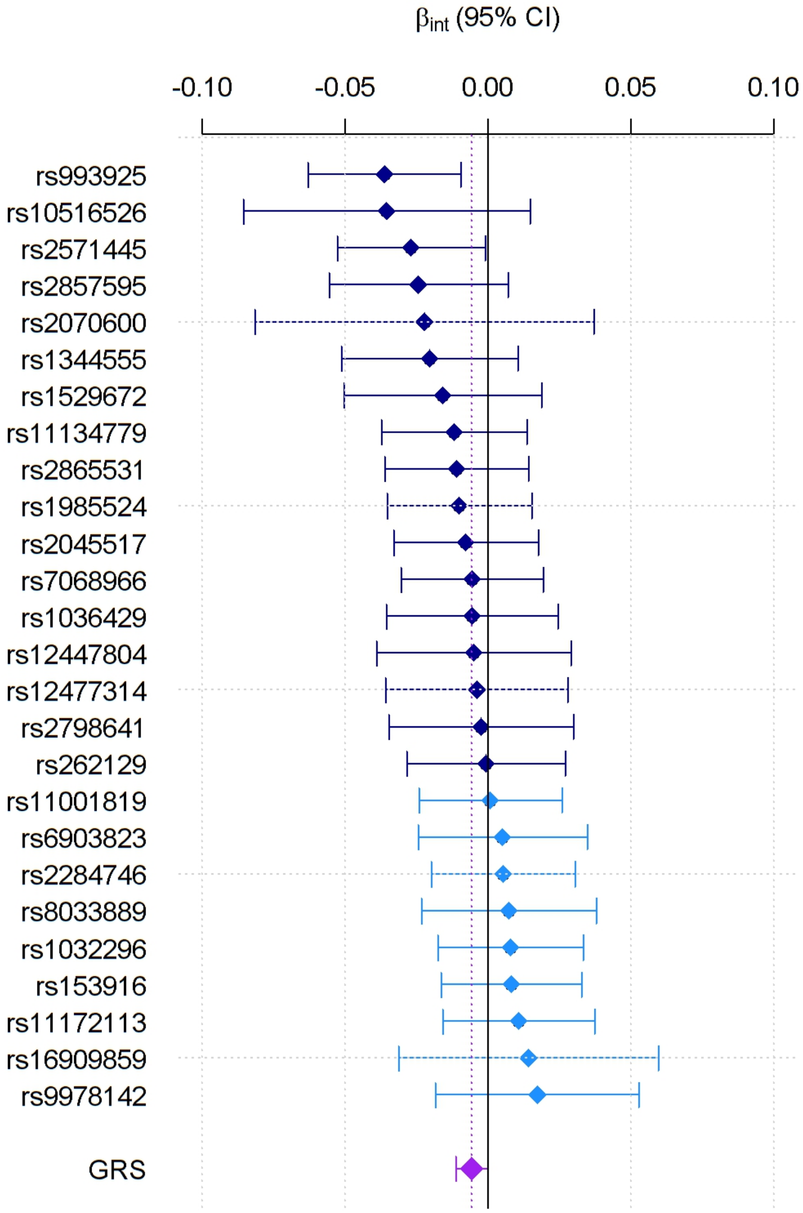

Supplementary Figure 5. Overview of the unweighted genetic risk score by smoking interaction effect on FEV_1.

Upper panel (A) presents the distribution of the unweighted genetic risk score (GRS, grey density plot) and the relationship between the unweighted GRS and standardized FEV_1 in ever smokers (red line) and never-smokers (black line). Lower panel (B) shows the excess relative risk (RR) of having FEV_1 in the lowest 1%, 5% and 20% of the population for ever smokers as compared to never smokers, stratified by GRS quintiles.

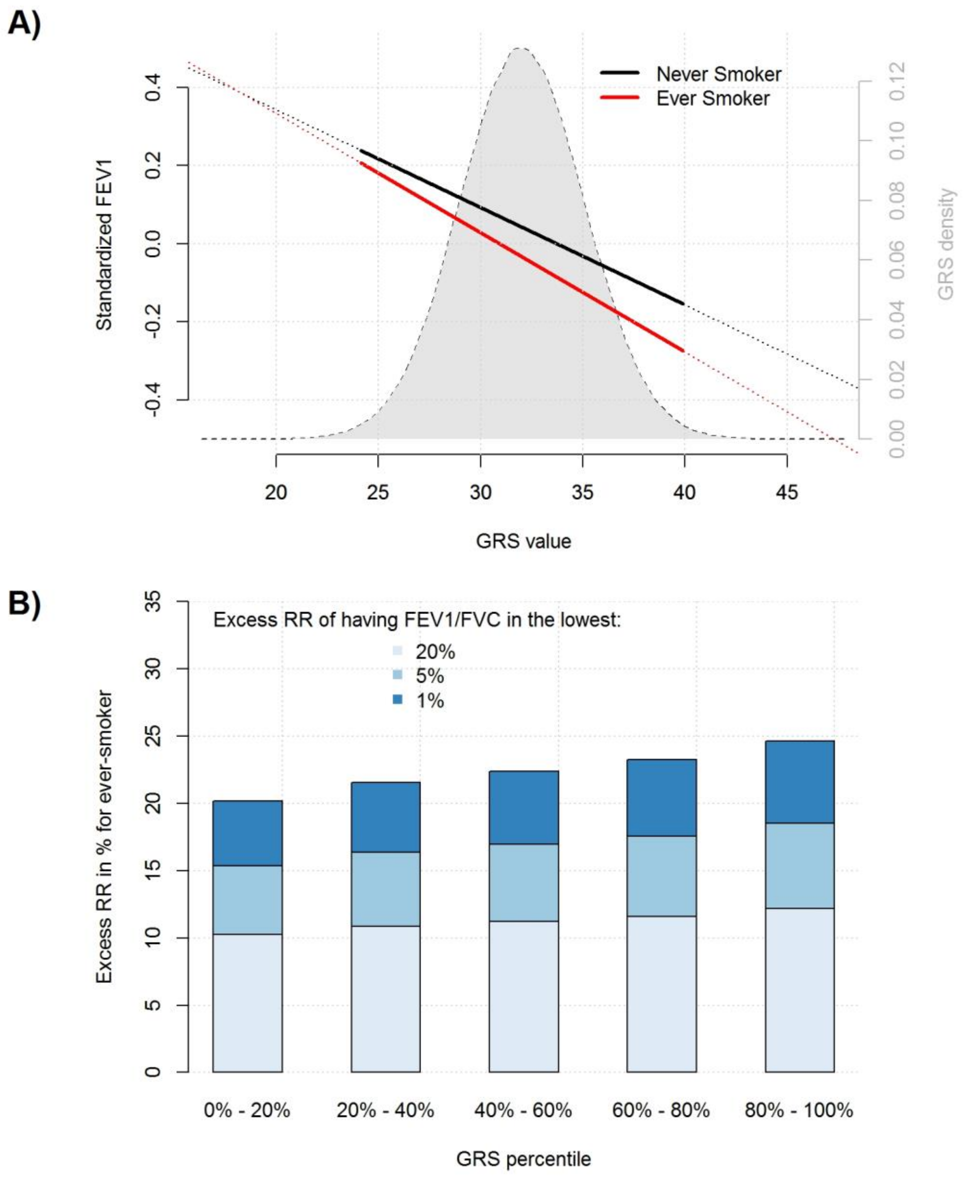

Supplementary Figure 6. Covariate-induced bias of interaction term in stratified analysis.

We simulated series of datasets where an outcome is defined as a function of a genetic variant, an exposure, and their interaction, and covariates present in unexposed or exposed individuals only. Upper panel presents the interaction effect estimated from the standard interaction test in the entire dataset (black) and from a two-steps stratified. The latter approach consisted of (i) normalizing the outcome using inverse-normal rank based transformation in each exposure strata separately, (ii) deriving marginal genetic effect in each strata separately, and (iii) inferring interaction effect from the marginal stratified result. When assuming a covariate effect in unexposed only (orange), the stratified approach shows overestimated interaction. Conversely, when assuming a covariate effect in exposed only (blue), interaction tend to be biased toward the null. Lower panel shows empirical statistical power observed for the three approaches.

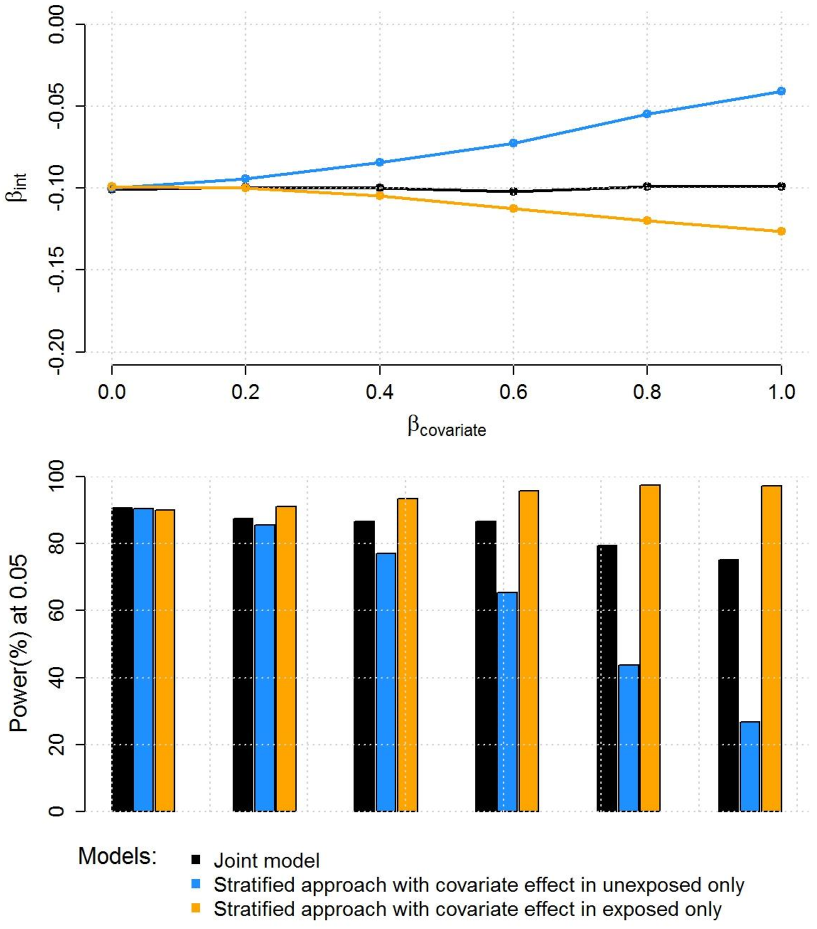

References

1. Hancock DB, Artigas MS, Gharib SA, et al. Genome-wide joint meta-analysis of SNP and SNP-by-smoking interaction identifies novel loci for pulmonary function. *PLoS genetics* 2012;**8**(12): e1003098.
2. Aschard H. A perspective on interaction effects in genetic association studies. *Genet Epidemiol* 2016.
3. Soler Artigas M, Wain LV, Miller S, et al. Sixteen new lung function signals identified through 1000 Genomes Project reference panel imputation. *Nature communications* 2015;**6**: 8658.
4. Hancock DB, Soler Artigas M, Gharib SA, et al. Genome-wide joint meta-analysis of SNP and SNP-by-smoking interaction identifies novel loci for pulmonary function. *PLoS genetics* 2012;**8**(12): e1003098.
5. Soler Artigas M, Loth DW, Wain LV, et al. Genome-wide association and large-scale follow up identifies 16 new loci influencing lung function. *Nature genetics* 2011 Nov;**43**(11): 1082-90.
